# Estrogenic regulation of skeletal muscle proteome: a study of premenopausal women and postmenopausal MZ cotwins discordant for hormonal therapy

**DOI:** 10.1111/acel.12661

**Published:** 2017-09-07

**Authors:** Eija K. Laakkonen, Rabah Soliymani, Sira Karvinen, Jaakko Kaprio, Urho M. Kujala, Marc Baumann, Sarianna Sipilä, Vuokko Kovanen, Maciej Lalowski

**Affiliations:** ^1^ Faculty of Sport and Health Sciences Gerontology Research Center University of Jyväskylä Jyväskylä Finland; ^2^ Medicum, Biochemistry/Developmental Biology Meilahti Clinical Proteomics Core Facility University of Helsinki Helsinki Finland; ^3^ Divisions of Rehabilitation Science and Physical Therapy Department of Rehabilitation Medicine Medical School University of Minnesota Minneapolis MN USA; ^4^ Institute for Molecular Medicine FIMM University of Helsinki Helsinki Finland; ^5^ Faculty of Sport and Health Sciences University of Jyväskylä Jyväskylä Finland

**Keywords:** estrogenic regulation, female muscle, functional annotation, hormone replacement therapy, label‐free protein quantitation, nano‐LC‐HD‐MS^E^

## Abstract

Female middle age is characterized by a decline in skeletal muscle mass and performance, predisposing women to sarcopenia, functional limitations, and metabolic dysfunction as they age. Menopausal loss of ovarian function leading to low circulating level of 17β‐estradiol has been suggested as a contributing factor to aging‐related muscle deterioration. However, the underlying molecular mechanisms remain largely unknown and thus far androgens have been considered as a major anabolic hormone for skeletal muscle. We utilized muscle samples from 24 pre‐ and postmenopausal women to establish proteome‐wide profiles, associated with the difference in age (30–34 years old vs. 54–62 years old), menopausal status (premenopausal vs. postmenopausal), and use of hormone replacement therapy (HRT; user vs. nonuser). None of the premenopausal women used hormonal medication while the postmenopausal women were monozygotic (MZ) cotwin pairs of whom the other sister was current HRT user or the other had never used HRT. Label‐free proteomic analyses resulted in the quantification of 797 muscle proteins of which 145 proteins were for the first time associated with female aging using proteomics. Furthermore, we identified 17β‐estradiol as a potential upstream regulator of the observed differences in muscle energy pathways. These findings pinpoint the underlying molecular mechanisms of the metabolic dysfunction accruing upon menopause, thus having implications for understanding the complex functional interactions between female reproductive hormones and health.

## Introduction

Skeletal muscle aging is characterized by progressive decline in muscle mass and function predisposing to sarcopenia, functional limitation, and metabolic dysfunction (Cruz‐Jentoft *et al*., 2010). Muscle aging is a multifactorial process affected by intrinsic factors such as endocrine changeover, neuronal remodeling, muscle regeneration deficiency and deterioration of cell death regulation, and extrinsic factors such as diet, physical activity, sedentariness, or other lifestyle choices (Demontis *et al*., 2013).

Up until loss of ovarian function at menopause, 17β‐estradiol (E_2_) is the most prominent biologically active circulating female sex hormone. Among fertile premenopausal women, the circulating serum concentration of E_2_ fluctuates from few tens of pmols L^−1^ to over 1000 pmol L^−1^ depending on a phase of the menstrual cycle (Laven & Fauser, 2006). Menopausal transition is characterized by irregular menstrual cycle leading eventually to amenorrhea, concomitantly with a shift in hormonal balance from higher E_2_ and lower follicle‐stimulating hormone (FSH) to constantly low E_2_ and high FSH levels (Harlow *et al*., 2012; Baber *et al*., 2016). Although sex differences in skeletal muscle metabolism and the role of ovarian hormones and benefits of postmenopausal hormone replacement therapy (HRT) in combating aging‐related decrements of muscle properties have gained a significant interest in last decades (e.g., Greising *et al*., 2009; Velders & Diel, 2013; Sipilä *et al*., 2015; Gheller *et al*., 2016), current knowledge base is overwhelmingly contributed by male studies with fewer input coming from studies concentrating on female‐specific aspects of aging.

The circulating female hormone pool is not the only hormonal pool affecting muscle properties. We have recently shown that intramuscular E_2_ concentration is associated with muscle strength and power, independently from the E_2_ concentration in the circulation (Pöllänen *et al*., 2015). The origin of intramuscular hormones and their exact molecular functions are currently unknown. Muscle tissue may directly respond to circulating E_2_ as well as uptake dehydroepiandrosterone (DHEA), testosterone, or other precursor hormones, to be converted to biologically active E_2_, which then contributes to the intracellular signaling. It is well documented that muscle cells express estrogen and androgen receptors enabling receptor‐dependent signaling and direct interactions with other pathways such as IGF‐1/Akt signaling (Olivieri *et al*., 2014). To gain a more comprehensive view on the responses of skeletal muscle to hormonal aging, multiple ‐omics approaches, including transcriptomics, proteomics, and metabolomics are desired.

Recently, a French research consortium identified 67 putative biomarkers of muscle aging by comparing muscle samples from groups of 56‐ and 78‐year‐old postmenopausal women (Gueugneau *et al*., 2014; Theron *et al*., 2014). Candidate proteins for muscle aging were classified into eight functional groups, including myofilaments and cytoskeleton, energy metabolism, detoxification, cytoprotection, signal transduction, proteostasis, proteolysis, and serum proteins. However, in these studies the role of hormone status on muscle aging was not investigated. To complement the previous investigations on aging female muscle proteome, we focused on possible differences evoked by menopause‐associated hormonal aging in adult premenopausal and middle‐aged postmenopausal women. In the current study, we investigated the age‐ and hormone status‐associated differences in female muscle proteome in relation to differences in muscle performance. We used skeletal muscle samples of premenopausal (30‐ to 34‐year‐old) women in comparison with postmenopausal (54‐ to 62‐year‐old) women who were either never exposed to or current HRT users. Moreover, we utilized a genetically controlled cotwin model to investigate the relationship between HRT and muscle proteome among postmenopausal women with, on average of 7‐year discordance, in the history of HRT use. Finally, potential differences due to different types of HRT, namely E_2_‐ or tibolone‐based product, were investigated. This approach enabled determination of differences in female muscle proteome that are associated with hormonal aging, that is, differences in age and E_2_ and pinpointing E_2_ as a major regulator of female muscle energy and cell death pathways.

## Results and discussion

Clinically and phenotypically characterized female muscle samples were lysed and subjected to data‐independent nano‐LC‐HD‐MS^E^ analysis, followed by protein identification and relative abundance quantitation using the Progenesis QI for Proteomics platform. Subsequently, functional characterization using Ingenuity Pathway Analysis (IPA) software was performed. We determined the proteomic differences and the affected molecular pathways due to differences in age and hormonal status as well as predicted upstream regulators of the affected pathways. Due to differences in the functional mechanism of estrogen‐based (E_2_‐HRT) and tibolone‐based (Tib‐HRT) HRT, the proteomics analysis was performed separately for these subgroups.

### Clinical characteristics of study participants

Study participants included six healthy 30‐ to 34‐year‐old premenopausal women and 18 women from nine pairs of 54‐ to 62‐year‐old postmenopausal monozygotic (MZ) cotwin sisters discordant for current HRT use (Ronkainen *et al*., 2009; Pöllänen *et al*., 2011). The clinical and gynecological characteristics are summarized in Table 1. Among the HRT using twin sisters, three used HRT medication which contained only estradiol and three a medication which included estradiol and progestogen forming the E_2_‐HRT group, while another three used tibolone‐containing HRT (Tib‐HRT group), known to possess estrogenic, progestogenic, and androgenic functions (Dubey *et al*., 2004; Hanifi‐Moghaddam *et al*., 2005). As estradiol‐only HRT and estradiol plus progestogen HRT are based on the utilization of estradiol as a main functional hormone, and there were no phenotypic differences between the users (data not shown), these two groups were combined to increase the statistical power of the proteomic analysis. All participants were in good self‐reported health, although some of them reported minor medical conditions such as allergies, musculoskeletal problems, migraine, or had hypertension with medication (Table 1). Furthermore, one postmenopausal woman had a history with malignant melanoma, for which she had undergone treatment for 15 years before the study onset.

**Table 1:** Clinical data of the study participants

	Premenopausal *n* = 6	Postmenopausal nonusers *n* = 9	Postmenopausal HRT users *n* = 9
Age (years)	31.8 ± 1.8	57.3 ± 2.2	57.3 ± 2.2
Health and lifestyle variables			
Number of chronic diseases or conditions			
Allergy (*n*)	4	2	1
Musculoskeletal problems (*n*)	4	4	3
Migraine (*n*)	3	1	0
Cancer history (*n*)	0	1	0
Hypertension with medication (*n*)	0	4	5
Current smokers (*n*)	2	1	1
Physical activity			
Sedentary (*n*)	0	1	1
Moderately active (*n*)	3	3	3
Active (*n*)	3	5	4
Gynecological data			
Age at first menstruation (years)	13.3 ± 2.0	12.9 ± 1.4	12.6 ± 1.0
Age at last menstruation (years)	Still menstruating	48.7 ± 4.2	50.7 ± 2.0
Number of participants with regular menstrual cycle (*n*)	6	No cycle	No cycle
Mean length of the cycle (days)	28.3		
Number of participants been pregnant (*n*)	4	8	7
Number of pregnancies per participant (*n*)	0–3	0–3	0–3
Number of participants having used hormonal contraceptive during past 10 years; but not within last 5 years (*n*)	3	1	1
Number of participants with ovariectomy and/or hysterectomy (*n*)	0	1	3
Number of participants reporting menopausal symptoms	0	9	7
Use of HRT	Never	Never	Currently
Duration of HRT use (years)			7.3 ± 3.3
Number of E_2_‐HRT users (*n*)			3
Number of E_2_+progestogen‐HRT users (*n*)			3
Number of Tib‐HRT users			3

Data are presented either as mean ± standard deviation or as total number of participants. HRT = hormone replacement therapy, E_2_ = 17β‐estradiol, Tib = tibolone.

The phenotype characteristics of the study participants are presented in Table 2. There were no significant differences in body composition between pre‐ and postmenopausal women, but according to handgrip strength measurements, premenopausal women were stronger than non‐HRT postmenopausal women. Premenopausal women were also more powerful than postmenopausal women regardless of the HRT status, and the HRT users had greater muscle power than their nonusing cotwins (Table 2). The expected menopause‐ and HRT status‐related differences were seen in systemic hormones status, namely in the circulating concentrations of E_2_, testosterone, luteinizing hormone (LH), and FSH (Table 2). As we have previously demonstrated (Pöllänen *et al*., 2015), there were no differences in intramuscular steroid hormone levels except for higher dihydrotestosterone (DHT) levels in postmenopausal HRT users and nonusers compared to premenopausal women.

**Table 2:** Phenotype characteristics of the study participants. Statistically significant *P*‐values are marked in bold

	Premenop. Women (*n* = 6)	Postmenop. nonusers (*n* = 9)	Postmenop. HRT users (*n* = 9)	*P*‐values		
				PRE vs. nonusers	PRE vs. HRT users	HRT‐ vs. nonusers
Body composition						
BMI (kg m^−2^)	23.1 ± 2.2	26.9 ± 4.6	25.4 ± 2.4	0.082	0.079	0.261
Waist circumference (cm)	80.8 ± 10.1	90.1 ± 10.1	85.5 ± 5.0	0.106	0.252	0.176
Hip circumference (cm)	97.0 ± 5.3	102.6 ± 7.9	99.8 ± 5.3	0.156	0.338	0.074
Body fat mass (kg)	15.7 ± 5.1	23.9 ± 8.9	19.8 ± 4.8	0.064	0.135	0.121
LBM (kg)	43.3 ± 3.3	44.9 ± 4.0	45.7 ± 2.6	0.437	0.146	0.527
Muscle performance						
Handgrip (N)	333.6 ± 45.7	258.9 ± 36.1	289.1 ± 57.0	**0.004**	0.134	0.218
Power (cm)	26.6 ± 5.7	12.6 ± 3.8	15.5 ± 2.8	**<0.001**	**<0.001**	**0.011**
Specific force (N cm^−2^)	10.1 ± 1.8	9.8 ± 1.7	9.4 ± 1.5	0.732	0.424	0.470
Systemic hormones^a^						
E_2_ (pmol L^−1^)	461.7 ± 308.2	26.0 ± 8.5	151.0 ± 233.9	**<0.001**	**0.008**	**0.018**
Testosterone (nmol L^−1^)	1.1 ± 0.4	0.8 ± 0.3	0.7 ± 0.2	0.224	**0.050**	0.374
DHT (pmol L^−1^)	468.3 ± 319.8	201.1 ± 110.8	268.9 ± 91.2	**0.026**	0.181	0.141
DHEAS (μmol L^−1^)	4.4 ± 1.6	3.2 ± 1.5	3.5 ± 1.7	0.066	0.088	0.374
LH (IU L^−1^)	9.6 ± 5.3	32.7 ± 15.6	28.6 ± 6.0	**0.001**	**0.001**	0.214
FSH (IU L^−1^)	6.2 ± 1.7	82.9 ± 20.3	54.9 ± 26.1	**<0.001**	**<0.001**	**0.015**
SHBG (nmol L^−1^)	54.2 ± 18.4	54.1 ± 23.8	54.3 ± 29.3	0.689	0.864	0.767
Intramuscular hormones^a^						
E_2_	1.3 ± 0.1	1.3 ± 0.3	1.2 ± 0.2	0.776	0.607	0.058
Testosterone	11.8 ± 1.8	12.3 ± 2.5	11.0 ± 1.6	0.689	0.529	0.086
DHT	0.5 ± 0.1	0.9 ± 0.2	0.8 ± 0.1	**<0.001**	**<0.001**	**0.011**
DHEA	70.1 ± 11.1	70.7 ± 14.7	61.9 ± 11.8	0.776	0.224	0.110

Data are presented as mean ± standard deviation. HRT = hormone replacement therapy, BMI = body mass index, LBM = lean body mass, E_2_ = 17β‐estradiol, DHT = dihydrotestosterone, DHEA(S)  = dehydroepiandrosterone (sulfate), LH = luteinizing hormone, FSH = follicle‐stimulating hormone, SHBG = sex hormone‐binding globulin.

a Due to nonlinear distribution of hormone variables, Mann–Whitney *U*‐test for independent samples was used to test significance of group means in PRE vs. nonuser and PRE vs. HRT user comparisons and Wilcoxon signed rank test for related samples to test significance of group means in HRT vs. nonuser comparison.

To investigate the potential cotwin differences in clinical characteristics between E_2_‐HRT and Tib‐HRT groups, the mean intrapair differences within both groups were calculated (Table 3). Among the E_2_‐HRT group, intrapair differences existed for muscle power, systemic E_2_, sex hormone‐binding globulin (SHBG), and for intramuscular DHT while among Tib‐HRT group a significant difference between the sisters was observed in specific muscle force. E_2_‐ and Tib‐HRT groups differed from each other in specific force and systemic E_2_, testosterone, and SHBG levels. These results further confirm that E_2_‐ and Tib‐HRT have different mechanisms of function, which subsequently leads to differences in the phenotype.

**Table 3:** The intrapair differences of postmenopausal twins. Statistically significant *P*‐values are marked in bold

	E_2_‐based HRT (*n* = 6 pairs)		Tibolone‐based HRT (*n* = 3 pairs)		Group comparison
	IPD	*P*‐value	IPD	*P*‐value	*P*‐value
Body composition					
BMI	−2.78 ± 3.88	0.140	1.07 ± 1.59	0.363	0.152
Waist circumference	−7.20 ± 10.32	0.148	0.67 ± 3.55.0	0.774	0.253
Hip circumference	−3.78 ± 4.83	0.114	−0.89 ± 0.92	0.236	0.354
Body fat mass	−6.82 ± 6.76	0.057	1.44 ± 3.97	0.594	0.097
LBM	−0.06 ± 3.79	0.972	2.45 ± 2.76	0.265	0.349
Muscle performance					
Handgrip	25.53 ± 69.32	0.408	39.30 ± 77.66	0.473	0.794
Power	3.58 ± 2.89	**0.029**	1.41 ± 1.20	0.179	0.262
Specific force	0.48 ± 0.94	0.268	−2.11 ± 0.53	**0.020**	**0.003**
Systemic hormones^a^					
E_2_	187.17 ± 275.10	**0.027**	0.67 ± 5.13	0.785	**0.024**
Testosterone	40.0 ± 95.71	0.462	−423.33 ± 268.39	0.109	**0.024**
DHT	0.03 ± 0.08	0.345	0.13 ± 0.17	0.276	0.381
DHEAS	−0.22 ± 2.01	0.917	1.44 ± 1.25	0.109	0.262
SHBG	25.08 ± 25.45	**0.028**	−49.40 ± 24.57	0.109	**0.024**
LH	−7.92 ± 10.09	0.116	3.73 ± 9.88	0.593	0.262
FSH	−30.98 ± 33.79	0.075	−22.2 ± 7.28	0.109	1.0
Intramuscular hormones^a^					
E_2_	−0.11 ± 0.28	0.399	−0.22 ± 0.13	0.109	0.167
Testosterone	−1.18 ± 2.28	0.345	−1.59 ± 0.82	0.109	0.381
DHT	−0.12 ± 0.17	**0.046**	−0.19 ± 0.13	0.109	0.381
DHEA	−5.78 ± 17.48	0.600	−14.63 ± 7.38	0.109	0.262

IPD = intrapair difference, E_2_ = 17β‐estradiol, HRT = hormone replacement therapy, BMI = body mass index, LBM = lean body mass, DHT = dihydrotestosterone, DHEA(S)  =  dehydroepiandrosterone (sulfate), LH = luteinizing hormone, FSH = follicle‐stimulating hormone, SHBG = serum hormone‐binding globulin.

a Due to nonlinear distribution of hormone variables, Wilcoxon signed rank test for related samples was used to test significance of intrapair difference (IPD = HRT user value – nonuser value) within twin pairs and Mann–Whitney *U*‐test for independent samples was used to test significance of IPD between groups.

### General characterization of female muscle proteome

Using nano‐LC‐HD‐MS^E^, we identified in total 1583 proteins of which 797 were quantified (Table S1, Supporting information) and subjected to further analysis by applying the following scheme: (i) Postmenopausal nonhormone users (non‐HRT; *n* = 9) were compared to premenopausal women (PRE; *n* = 6), in order to identify hormonal aging‐associated differences at low E_2_ background; (ii) postmenopausal E_2_‐HRT users (*n* = 6) were compared to PRE (*n* = 6), in order to identify hormonal aging‐associated differences at E_2_‐supplemented background; and (iii) to identify HRT‐use‐associated differences at genetically controlled, same age background, two separate comparisons were made: (iii*‐*a) Postmenopausal E_2_‐HRT women (*n* = 6) were compared to their nonusing cotwins (*n* = 6); and (iii‐b) postmenopausal Tib‐HRT users (*n* = 3) were compared to their nonusing cotwins (*n* = 3).

Using this strategy and applying stringent filtering (│fold change (FC) > 1.5│, *P* < 0.05, ≥ 2 unique peptides observed by nano‐LC‐HD‐MS^E^) to identify differentially expressed proteins (DEPs, Table S2, Supporting information), we identified hormonal aging‐associated differences in the relative abundance of 114 proteins at low E_2_ background (non‐HRT vs. PRE) and 151 proteins at E_2_‐supplemented background (E_2_‐HRT vs. PRE). Furthermore, E_2_‐HRT users differed from their non‐HRT cotwins in the relative abundance of 53 proteins, while Tib‐HRT users differed from their non‐HRT cotwins in the relative abundance of 95 proteins.

**Figure 1:**
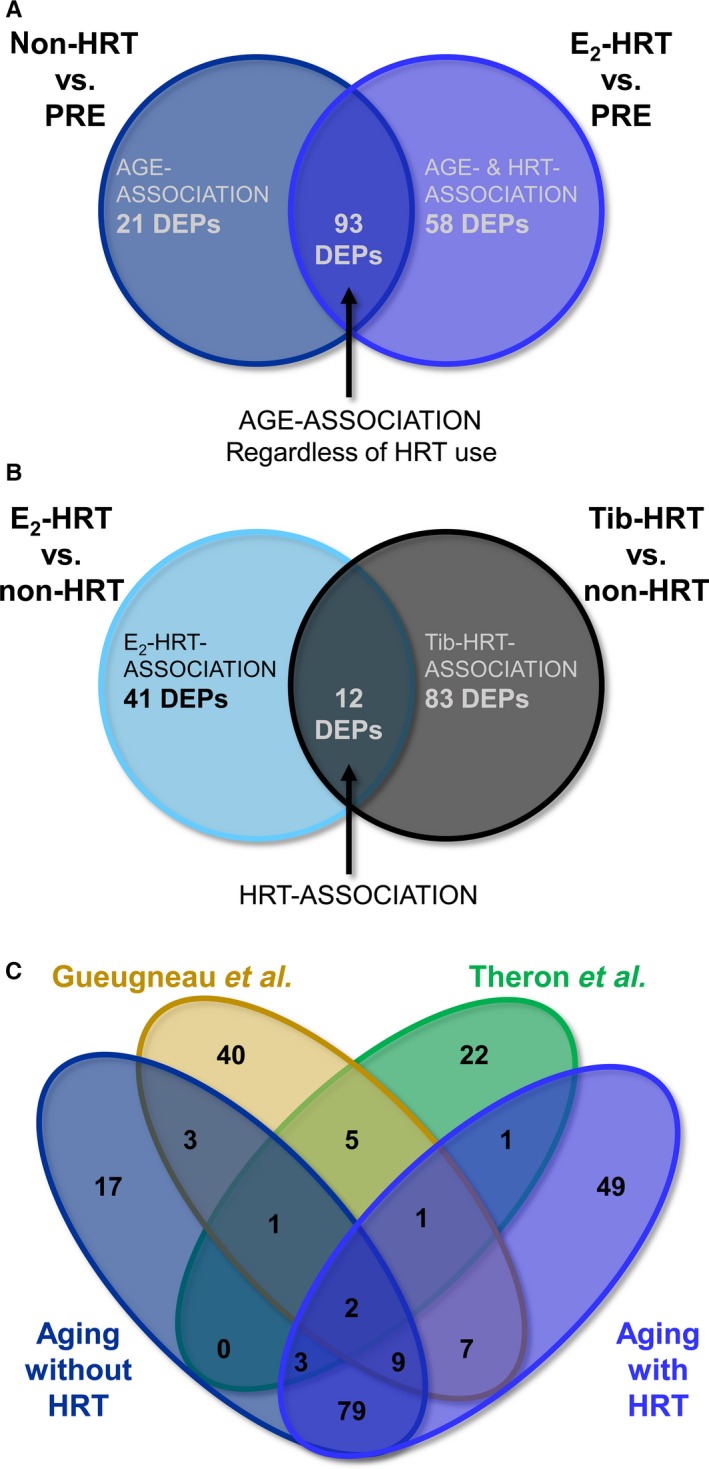
Characterization of female muscle proteome. (A) Venn diagram comparison of differentially expressed proteins (DEPs) in relation to the differences in age with or without hormone replacement therapy (HRT) treatments. (B) Venn diagram comparison of DEPs in relation to differences in the HRT specimen (E_2_‐based or tibolone‐based). (C) Comparison of quantitative proteomics data with literature findings. Twenty‐eight proteins were identified in previous proteomic studies by Théron *et al*. and Gueaugneau *et al*., while 93 of the proteins were novel. PRE = premenopausal group, non‐HRT = postmenopausal women not using HRT, E_2_‐HRT = postmenopausal women using 17β‐estradiol‐based HRT, Tib‐HRT =  postmenopausal women using tibolone‐based.

The identified DEPs were compared in order to identify unique and shared molecular processes related to age‐ and HRT‐associated differences (Fig. 1). When postmenopausal non‐HRT women or HRT using women were compared to the premenopausal women, 93 shared proteins were identified in both comparisons indicating that the use of HRT does not affect the age‐related differences in the abundance of these proteins (Fig. 1A, Table S3, Supporting information). Moreover, 21 proteins were unique to non‐HRT vs. PRE comparison, and 58 to HRT users vs. PRE comparison. Furthermore, we identified 12 HRT‐associated proteins regardless the HRT type, 41 proteins specific to E_2_‐HRT, and 83 proteins specific to Tib‐HRT (Fig. 1B, Table S3, Supporting information).

As a part of our validation scheme, we compared our results and the findings of previous muscle proteomics studies on postmenopausal aging between 56 and 78 years of age (Gueugneau *et al*., 2014; Theron *et al*., 2014). Among the age‐associated DEPs (114 proteins at low E_2_ background and 151 proteins at E_2_‐supplemented background), 27 proteins have been previously described (Fig. 1C, Table S3, Supporting information). In addition to previously known age‐associated DEPs, we identified 145 novel proteins to be associated with pre‐ to postmenopausal hormonal aging occurring between in 30–34 and 54–62 years of age. Seventy‐nine of them were present in both aging conditions with or without HRT. This indicates that although we were able to identify several novel muscle proteins with abundance influenced by aging, 54% of them were not sensitive to the use of HRT and, thus, potentially are not affected by the circulating level of E_2_. However, the use of same younger control group in both comparisons may decrease variation between comparisons and, thus, increase the similarity of the findings.

### Functional muscle proteomics

**Figure 2:**
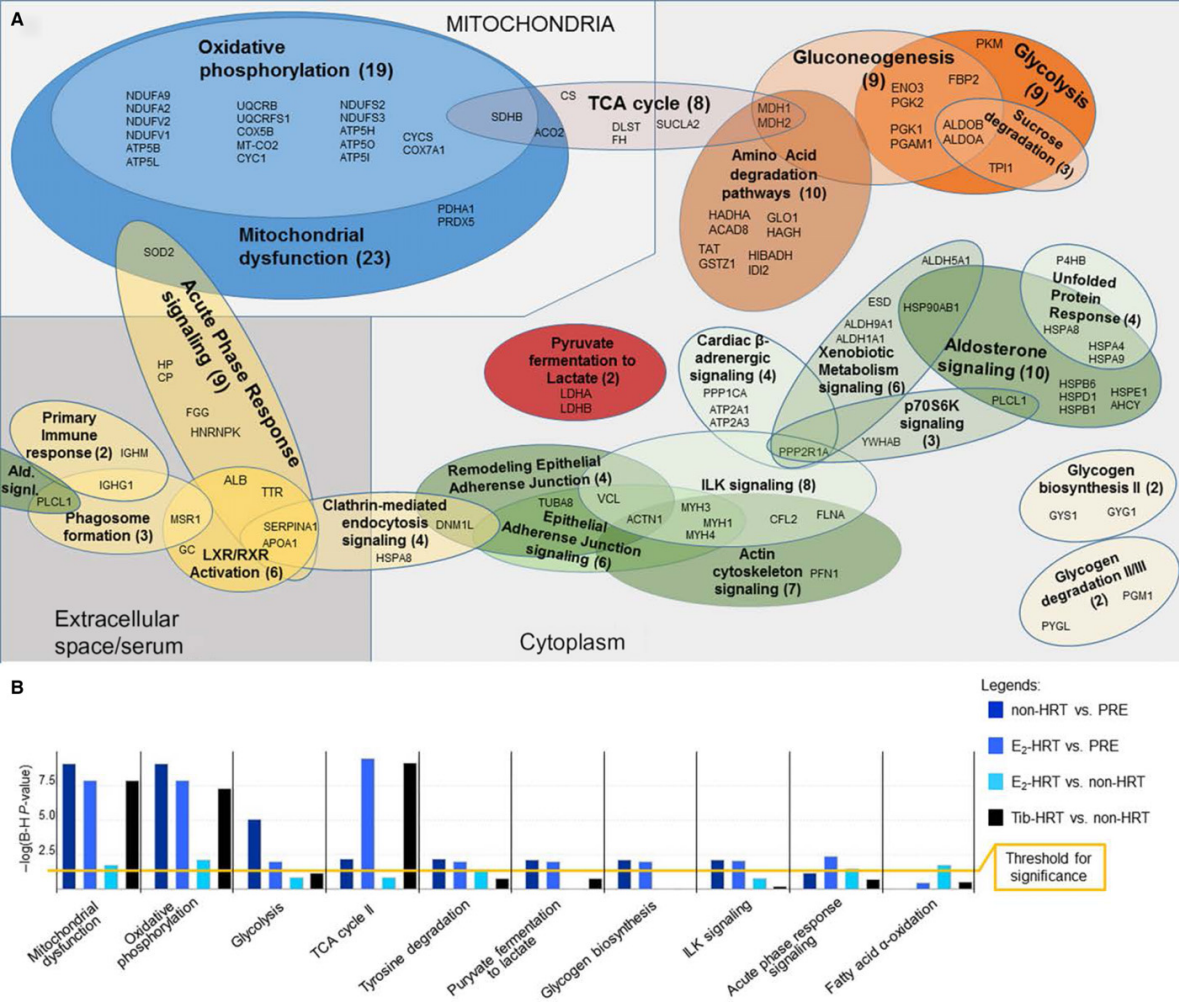
Functional analysis of the muscle proteome. (A) Functional distribution of differentially expressed proteins (DEPs) contributing to the canonical pathways (B) revealed substantial overlap and pinpointed the major differences in the expression to affect mitochondria (e.g., oxidative phosphorylation), cytoplasmic energy metabolism and various cellular signaling modules. (B) Canonical pathways analysis. Ten pathways with most significant Benjamini–Hochberg corrected *P*‐values are presented. PRE = premenopausal group, non‐HRT = postmenopausal women not using HRT, HRT = hormone replacement therapy, E_2_‐HRT = postmenopausal women using 17β‐estradiol‐based HRT, Tib‐HRT =  postmenopausal women using tibolone‐based, B‐H = Benjamini–Hochberg correction.

The lists of DEPs were subsequently functionally annotated with IPA. In total, 39 canonical pathways were found to be significantly affected (Benjamini–Hochberg adjusted *P* value, B‐H *P* < 0.05) in one or more comparisons (Table S4, Supporting information). Closer characterization of the identified pathways and their contributing proteins demonstrated grouping in three major functional clusters associated with mitochondrial functions, cytoplasmic energy metabolism, and cellular signaling linked with immune response (Fig. 2A).

Among the statistically significantly affected (B‐H *P* < 0.05) canonical pathways, ten with highest B‐H *P* values are presented in the Fig. 2B. Majority of the affected pathways were shared between all four conditions, but for E_2_‐HRT vs. non‐HRT, the associations were in most cases statistically nonsignificant. This may indicate that the use of E_2_‐HRT suppresses some of the aging effects. This finding is in line with our earlier transcriptomic study (Pöllänen *et al*., 2007), which demonstrated that the use of E_2_‐HRT mitigates genome‐wide changes in postmenopausal women's muscle gene expression. Significant age associations were found for 18 pathways without E_2_‐supplementation and for 18 pathways with E_2_‐supplementation in contrast to 19 pathways identified in E_2_‐HRT vs. non‐HRTs comparison, and seven pathways in Tib‐HRT vs. non‐HRT comparison (Fig. 2B, Table S4, Supporting information). The potential protective role of HRT was not always associated with the use of Tib‐HRT. For example, glycolysis, mitochondrial dysfunction, aspartate degradation, and tricarboxylic acid (TCA) cycle pathways were significantly enriched in the Tib‐HRT vs. non‐HRT in comparison with E_2_‐HRT vs. non‐HRT (Fig. 2B).

### Upstream regulators and downstream functions associated with female muscle

In order to identify potential upstream effectors behind the observed protein level differences and to further anticipate which downstream functions are affected, we subsequently performed up‐ and downstream analyses with IPA. These analyses were based on the Ingenuity^®^ Knowledge Base regarding the associations between the multitude of upstream effectors and their targets which is combined with the measured observations, that is, our lists of DEPs.

**Figure 3:**
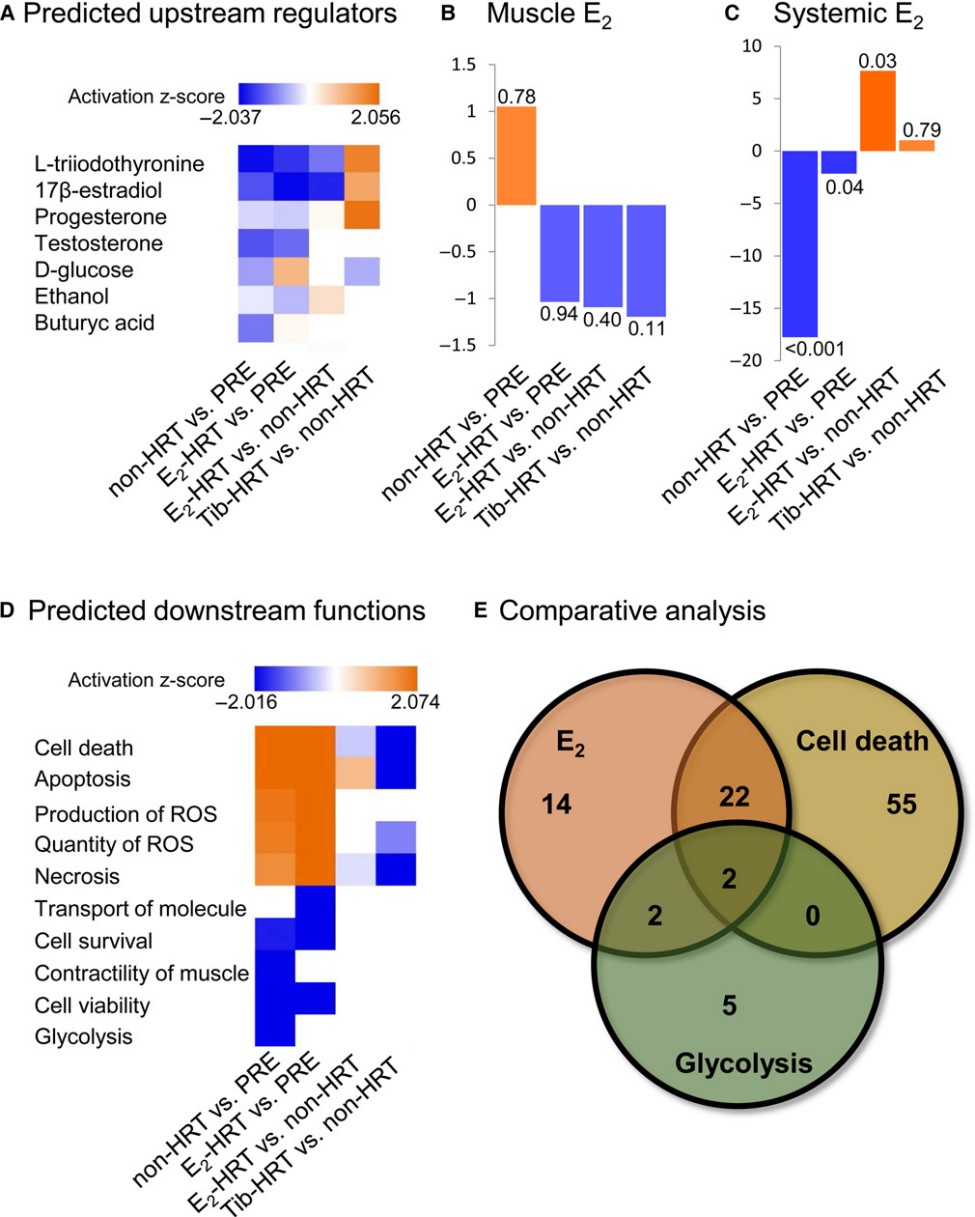
Upstream and downstream functions associated with female muscle aging with or without hormone replacement. (A) The upstream regulator analysis predicted 17β‐estradiol (E_2_) as one of the key upstream regulators behind the observed differences in muscle protein expression. (B) Fold change (FC) values of E_2_ measured from the muscle biopsy samples. The numbers at the end of the bars are *P*‐values. (C) Fold change (FC) values of the systemic E_2_ measured from the serum samples of pre‐ and postmenopausal women. The numbers at the end of the bars correspond to *P*‐values. (D) The downstream effect analysis predicted cell death, glycolysis, and muscle function pathways to be affected based on the observed differences in muscle protein expression. ROS = reactive oxygen species. (E) Comparative analysis of E_2_‐, cell death‐ and glycolysis‐associated differentially expressed proteins. IPA predicts cell death and glycolysis to be among the most affected downstream processes while E_2_ was predicted as an upstream regulator. However, the contributing DEPs for this prediction do not completely overlap. PRE = premenopausal women, non‐HRT = postmenopausal women not using HRT, HRT = hormone replacement therapy, E_2_‐HRT = postmenopausal women using 17β‐estradiol‐based HRT, Tib‐HRT = postmenopausal women using tibolone‐based HRT. The number of contributing DEPs is indicated.

**Figure 4:**
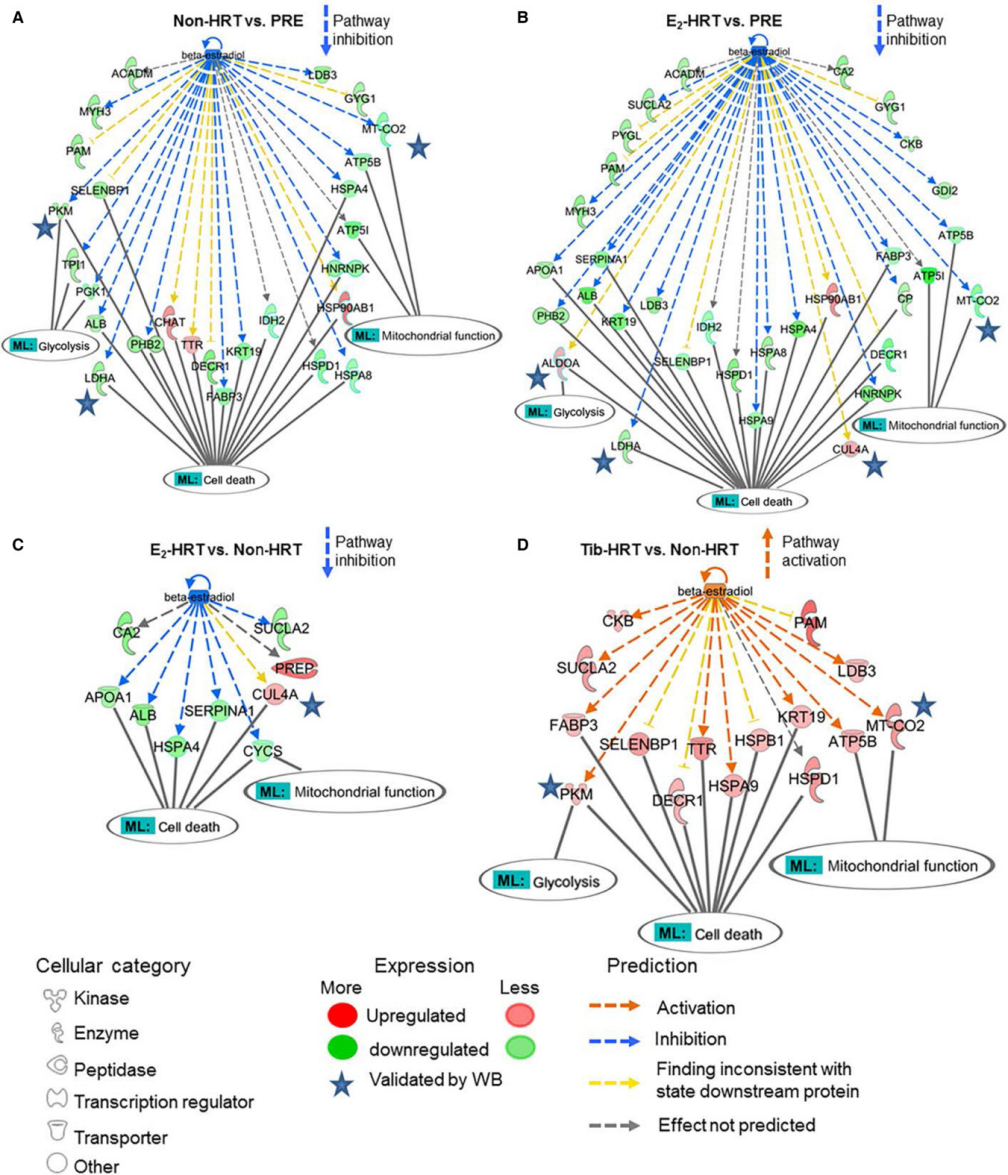
Comparative analysis of 17β‐estradiol (E_2_) regulated differentially expressed proteins (DEPs). (A) The DEPs identified in postmenopausal women without hormone replacement (HRT) in comparison with premenopausal women (non‐HRT vs. PRE). (B) Comparison of E_2_‐regulated DEPs among postmenopausal E_2_‐HRT users and premenopausal women (E_2_‐HRT vs. PRE). (C) Comparison of the E_2_‐regulated DEPs in postmenopausal E_2_‐HRT users and their nonusing cotwins (E_2_‐HRT vs. non‐HRT). (D) E_2_‐regulated DEPs in the postmenopausal Tib‐HRT users in comparison with their nonusing cotwins (Tib‐HRT vs. non‐HRT). The DEPs associated with the predicted downstream functions related to cell death and glycolysis as well as associated with mitochondrial functions are presented. Blue stars indicate DEPs with expression change validated by semi‐quantitative immunoblotting. ML = my lists are assembled by including all the observed DEPs involved in the corresponding pathways in any of the four conditions. ML: cell death and ML: glycolysis, originate from the analysis presented in the Fig. 3D, while ML: mitochondrial functions from the canonical pathways oxidative phosphorylation and mitochondrial dysfunction presented in the Fig. 2B. Arrows indicate the predicted inhibition or activation of the entire cascade.

Such analyses pinpointed E_2_ (17β‐estradiol) as one of the putative upstream effectors (Fig. 3A). Based on the observed alteration in muscle protein abundancies, IPA predicted E_2_ levels to be downregulated in all four comparisons except in Tib‐HRT vs. non‐HRT cotwins. To validate this prediction, E_2_ concentration was measured from muscle and serum samples in an independent set of experiments. The measured E_2_ concentration was not statistically different between groups in the muscle (Table 1 and Fig. 3B); however, the concentration of circulating, systemic E_2_ in the serum followed the IPA predictions in three of four conditions (Table 2 and Fig. 3C). The IPA prediction failed to reflect the order of E_2_ difference only in the E_2_‐HRT vs. non‐HRT comparison, in which the measured E_2_ level was higher in the E_2_‐HRT users as compared to the nonusers although IPA algorithm predicted it to be downregulated. E_2_‐HRT and non‐HRT women are MZ cotwins, which makes them very similar to each other. IPA cannot consider the cotwin design in the analysis; thus, the low genetic variance between groups may violate the correct prediction in the IPA's upstream analysis. However, several E_2_‐associated DEPs were identified for each group comparison (Fig. 4A–D).

The predicted downstream functions pointed to cell death and glycolytic pathways (Fig. 3D), which complements the canonical pathways analyses identifying glycolysis/gluconeogenesis as well as oxidative phosphorylation/mitochondrial dysfunctions among others to be affected by aging and use of HRT (Fig. 2). To get further insight and to verify that E_2_ is one of the key players for the predicted change in downstream cellular functions, we compared the proteins known to be associated with E_2_ (Fig. 4A–D) with proteins contributing to the cell death and glycolysis pathways (Fig. 3E), and identified 22 shared proteins with E_2_ and cell death pathways, two associated with E_2_ and glycolysis and two shared between all three lists. These results are supported by the measured muscle phenotype observations (Tables 2 and 3), which show uppermost muscle performance with highest levels of circulating E_2_, that is, in the premenopausal women and lowest muscle performance among non‐HRT women who concomitantly had the lowest circulating E_2_ level. Furthermore, these results indicate that aging‐associated decrements in muscle function may be mediated by cell death and glycolytic pathway‐associated DEPs and to be partially E_2_‐regulated.

### Functional validation of differentially expressed protein targets associated with estradiol

To complement and functionally validate the bioinformatics analyses, which identified E_2_ as significant upstream regulator of the muscle functions, we performed *in vitro* experiment with human muscle primary cells. Muscle progenitor cells were induced to differentiate from single nucleated myoblasts to multinucleated myotubes, which were then exposed to the E_2_. To follow the observed differences in the MS^E^ analyses on the cellular level, we confirmed the expression of selected E_2_‐associated DEPs by semi‐quantitative Western blotting from the myotubes exposed to mock conditions, and to 10 nm E_2_ or 100 nm E_2_ for 6 h.

We selected two DEPs predicted to be upregulated with lower E_2_ (non‐HRT vs. PRE and E_2_‐HRT vs. PRE) for validation. These proteins were aldolase fructose‐bisphosphate A (ALDOA) and cullin 4A (CUL4A), with important functions in energy pathways and in the regulation of cell cycle. ALDOA is a glycolytic enzyme that catalyzes the reversible conversion of fructose‐1.6‐bisphosphate to glyceraldehyde 3‐phosphate and dihydroxyacetone phosphate (Hittel *et al*., 2005), while CUL4A is E3 ubiquitin ligase with oncogenic effects through modulation of cell growth and immune response (Saucedo‐Cuevas *et al*., 2014). Furthermore, three DEPs predicted to be downregulated with lower E_2_ were also selected for semi‐quantitative analysis. These included lactate dehydrogenase A (LDHA), mitochondrial cytochrome c oxidase II (MT‐CO2), and muscle pyruvate kinase (PKM), which all have connections to muscle energy metabolism. Muscle LDHA catalyzes the conversion of lactate to pyruvate in the final step of anaerobic glycolysis (Kolappan *et al*., 2015). MT‐CO2 is the component of mitochondrial respiratory chain. Mutations in MT‐CO2 lead to MELAS, a multisystem disorder characterized by mitochondrial myopathy, lactic acidosis, and stroke‐like episodes followed by seizures, recurrent headaches, and muscle weakness (Rossmanith *et al*., 2009). The expression of PKM, which catalyzes the rate‐limiting final step in glycolysis, has been shown to be induced by E_2_ having critical role in metabolic reprogramming of proliferating cells toward aerobic glycolysis (Salama *et al*., 2014). Taken together, ALDOA, PKM, and LDHA have shared functions in glycolysis and cell death pathways while CUL4A and MT‐CO2 play a role in cell death.

**Figure 5:**
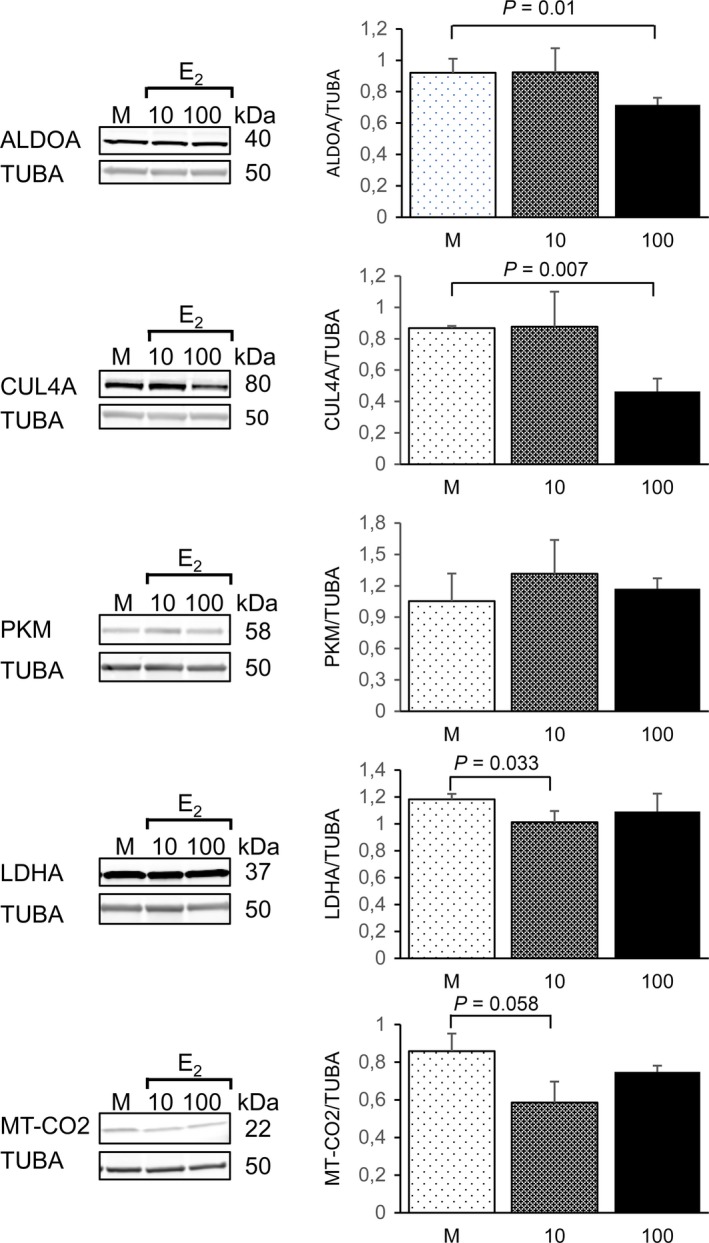
Validation of the proteomic results by semi‐quantitative Western blotting in primary human muscle cells. Representative images of the Western blots from cell culture experiments with human muscle primary cells. Quantification of the immunoblots was performed with three independent cell experiments exposed to the statistical testing using independent samples *t*‐test. Data are presented as mean ± standard deviation. M = mock, 10 = 10 nm E_2_, 100 = 100 nm E_2_.

The immunoblotting with ALDOA and CUL4A antibodies demonstrated a statistically significant change in protein expression upon treatment with 100 nm E_2_ (mock vs. E_2_; FC = 1.3, *P* = 0.01 for ALDOA and FC = 1.9, *P* = 0.007 for CUL4A), corroborating the results of HD‐MS^E^ (Fig. 5). Similarly, to the result of proteomic survey, immunoblotting with PKM antibody, which was predicted to be downregulated by IPA, demonstrated a trend for downregulation (mock vs. low E_2_; FC = −1.3); however, the difference between treatments was not statistically significant. Upon 10 nm E_2_ treatment, LDHA expression increased by 1.2‐fold (*P* = 0.033), while MT‐CO2 by 1.5‐fold, which however was not statistically significant. The observed discrepancy for LDHA and MT‐CO2 with corresponding proteomics measurements indicates that either longer E_2_ exposure time might be required to downregulate LDHA and MT‐CO2 expression in the muscle primary cells or other, yet unidentified factors, which are not represented *in vitro*, play a role in this process in the skeletal muscle tissue.

## Concluding remarks

Female aging is characterized by menopausal change in sex steroid hormones concomitant to increase in aging‐related decrements in skeletal muscle performance that can be attenuated by HRT use (Sipilä *et al*., 2015). The molecular mechanisms underpinning menopausal decrements in muscle function are poorly known. Our label‐free proteomic analyses have resulted in the quantification of 797 muscle proteins. Among those identified, 27 have been formerly investigated in large‐scale female muscle proteome studies (Gueugneau *et al*., 2014; Theron *et al*., 2014), while 145 were now described for the first time to our knowledge. Among the identified novel DEPs, 79 were measured in both aging conditions, with or without HRT, indicating them to be age‐responsive and insensitive to the use of HRT. In addition, 17 novel proteins were differentially expressed in postmenopausal women without HRT and 49 with HRT in comparison with the premenopausal women.

The major canonical pathways found to be differentially regulated included mitochondrial dysfunction, oxidative phosphorylation, glycolysis, and TCA‐cycle, strong indicators for affected energy metabolism. The major biological processes predicted to be affected were related to cell death, apoptosis, and cell survival, as well as contractility of the muscle and glycolysis. Furthermore, E_2_ was predicted to be an upstream regulator of these processes, which we confirmed by exposing myotubes to E_2_ *in vitro*. Previous experimental animal studies have implicated E_2_ in mitochondrial functions and metabolic homeostasis in skeletal (Capllonch‐Amer *et al*., 2014; Cavalcanti‐de‐Albuquerque *et al*., 2014; Ribas *et al*., 2016), and cardiac muscle (Rattanasopa *et al*., 2015; Sbert‐Roig *et al*., 2016). In addition, previous studies have shown E_2_ to exert anti‐apoptotic effects in muscle progenitor cells by improving mitochondrial function (La Colla *et al*., 2013).

Being in line with previous experimental studies, our study suggests, for the first time at human proteome level, that E_2_ is a major regulator of human skeletal muscle signaling in women. After menopause, when ovarian E_2_ production is ceased, the prevalence of cardio‐metabolic diseases increases. Our result that different trajectories of the energy pathways in the skeletal muscle may be regulated by E_2_ provides candidate molecules as key targets for future interventions to prevent or treat postmenopausal metabolic dysregulation. Further studies should focus on validation of the pathways identified in this study, to corroborate E_2_ as their direct upstream regulator.

## Experimental procedures

### Subjects and study design

The participants represented part of the SAWEs study, for which the details of recruitment and data collection have been reported earlier (Ronkainen *et al*., 2009; Pöllänen *et al*., 2011). The current study utilizes data and skeletal muscle (*m. vastus lateralis*) samples of six healthy premenopausal women and 18 healthy women from nine pairs of postmenopausal monozygotic cotwin sisters. Premenopausal women were recruited by postal enquiry sent to two thousand women randomly selected from the entire 30‐ to 40‐year‐old age cohort (born in 1967–1977) living in the City of Jyväskylä, Finland. Among the respondents, 59 were eligible after consideration of exclusion criteria (i.e., ongoing or previous (past 5 years) use of hormonal contraceptives, pregnancy, or breastfeeding). Six (10%) were selected at random for the current proteomic study (PASW Statistical Software, SPSS Inc., IBM, IL, USA). Postmenopausal women were recruited from the Finnish Twin Cohort (Kaprio & Koskenvuo, 2002). Postmenopausal twin pairs aged 54–62 years were asked to participate in SAWEs study if the other cosister had never used HRT and the other was a current HRT user. Total of 15 monozygotic twin pairs met the inclusion criteria and were willing to participate as described earlier (Ronkainen *et al*., 2009). Twin pairs were discordant for the HRT including nine non‐HRT users and their nine cotwins currently using HRT. This study was approved by the Local Ethical Committee of the Central Finland Hospital District (E0606/06) and performed following guidelines of the Helsinki Declaration and good clinical and scientific practice. All study participants provided written informed consent.

Study participants went through a medical examination during which a physician confirmed their general health status, gynecological history, absence of possible chronic or acute illnesses, and use of medication. Data on current and past smoking, alcohol consumption, and physical activity habits were collected with a standard questionnaire. Anthropometric and body composition measurements were performed between 07:00 and 10:00 after overnight fasting and were after standardized breakfast followed by muscle strength and power measurements. Body weight was measured with beam scale and height with a stadiometer and body mass index (BMI) calculated (weight, [kg]/height [m^2^]). Waist circumference was measured midway between *spina iliaca superior* and the lower rib margin and hip circumference at the level of the greater trochanters. Body fat mass and lean body mass (LBM) were measured with a multifrequency bioelectrical impedance analyzer (InBody 720; Biospace, Seoul, Korea). Maximal handgrip force was measured with an adjustable dynamometer chair (Good Strength, Metitur, Palokka, Finland). The explosive lower body muscle power, that is, ability to produce force as quickly as possible, was assessed with counter movement jump on a contact mat. Maximal isometric knee extension force was measured in a sitting position using an adjustable dynamometer chair (Good Strength, Metitur, Palokka, Finland). In all measurements, three to five trials were allowed and the maximal performance was accepted as the result. Quadriceps muscle cross‐sectional area was assessed from the computed tomography scans (Siemens Somatom Emotion Scanner; Siemens, Erlangen, Germany) and used to calculate specific force as maximal isometric knee extension force divided by muscle cross‐sectional area (Pöllänen *et al*., 2011).

### Blood and skeletal muscle samples and hormone assessments

Blood samples were taken between 08:00 and 10:00 h in standardized fasting conditions at supine position. For premenopausal women, samples were collected during 1‐5 days of estrous cycle. Muscle biopsies were taken following blood sampling from the mid‐part of the *m. vastus lateralis*. Visible blood, fat, and connective tissue were removed before the biopsy sample was mounted on the cork with Tissue Tek Optimal Cutting Temperature compound (OCT, Sakura; Alphen aan dae Rijn, the Netherlands), frozen rapidly in 2‐methylbutane (Sigma‐Aldrich Corporation, St. Louis, MO, USA) precooled to −160 °C in liquid nitrogen, and stored at −80 °C until used for protein analysis. Serum and muscle hormone assessments were carried out as in (Pöllänen *et al*., 2011). Briefly, serum concentrations of sex hormone‐binding globulin (SHBG), dihydroepiandrosterone sulfate (DHEAS), follicle‐stimulating hormone (FSH), and luteinizing hormone (LH) were measured using solid‐phase, chemiluminescent immunometric assays (Immulite 1000, Diagnostic Products, Los Angeles, CA, USA). Serum 17β‐estradiol (E_2_) was determined using an extraction radioimmunoassay, while testosterone and dihydrotestosterone (DHT) were measured separately using LC‐MS/MS. Muscle samples were homogenized on ice in Tissue Extraction Reagent I (Invitrogen, Carlsbad, CA, USA) supplemented with protease and phosphatase inhibitors. The amount of total soluble protein was determined using Pierce BCA Protein Assay‐kit (Thermo Scientific, Rockford, IL, USA). Muscular E_2_, testosterone, DHT, and DHEA concentrations were determine using ELISA tests (IBL‐international, Hamburg, Germany) from 1:10 diluted muscle supernatants in duplicates. The concentrations of muscular hormones were expressed as nmol × g^−1^ soluble muscle protein.

### Sample preparation and proteolytic digestion of proteins from muscle samples

Frozen muscle samples were thawed on ice, detached from cutting block, and submersed in milliQ water to wash excess amounts of OCT. Subsequently, they were dipped in 4% SDS/0.1 m Tris pH 8/0.05 m DTT and homogenized at room temperature using tissue homogenizer. The homogenate was heated for 10 min at near 100 °C and cleared by centrifugation at 30 000 *g* for 15 min. The protein concentration was determined using the nanodrop technique. 10 μg of total protein was digested, using modified FASP protocol (Scifo *et al*., 2015). In brief, the lysate buffer was exchanged by washing it several times with 8 m urea, 0.1 m Tris, pH 8 (urea buffer, UB). The proteins were alkylated with 50 mm iodoacetamide in UB, after washing out the DTT‐containing solution. 1:50 w/w of lysine‐C endopeptidase (Wako) was added in about 4 m urea/0.1 m Tris pH 8 and incubated at room temperature overnight. The peptide digests were collected by centrifugation, and trypsin solution was added in a ratio of 1:50 w/w in 50 mm ammonium bicarbonate. As before, the digests were collected and combined. The peptide samples were cleaned using C18—reverse phase ZipTip™ (Millipore), resuspended in 1% TFA and sonicated in water bath for 1 min.

### Liquid chromatography high‐definition tandem mass spectrometry

The samples were analyzed in randomized order. 300 ng of digested proteins/replicate (three technical replicates per sample) was used in nano‐LC‐HD‐MS^E^ analysis. The nano‐LC‐HD‐MS^E^ analyses were performed as described (Mäkelä *et al*., 2016; Tikka *et al*., 2016). Briefly, the peptides were separated by nanoAcquity UPLC system (Waters) equipped with a trapping column (5 μm Symmetry C18 180 μm × 20 mm C18 reverse phase, Waters), and followed by an analytical C18 BEH130 reversed‐phase column (75 μm × 250 mm, particle size 1.7 μm; Waters) in a single pump trapping mode. After trapping, the peptides were separated with a linear gradient of 3–35% of solution B (0.1% formic acid/acetonitrile), for 90 min at a flow rate 0.3 μL min^−1^ and stable column temperature of 35 °C. Each sample run was followed by two empty runs to wash out any remaining peptides from previous runs. The samples were run in ion mobility‐assisted data‐independent analysis mode (HD‐MS^E^), in a Synapt G2‐S mass spectrometer (Waters), by alternating between low collision energy (6V) and high collision energy ramp in the transfer compartment (20–45 V) and using 1 s cycle time. The separated peptides were detected online with mass spectrometer, operated in positive, resolution mode in the range of *m/z* 50–2000 amu. 150 fmol μL^−1^ of human [Glu^1^]‐fibrinopeptide B (Sigma) in 50% acetonitrile/0.1% formic acid solution at a flow rate of 0.3 μL min^−1^ was used for a lock mass correction, applied every 30 s.

### Database mining of proteomics data

Relative quantification between samples using precursor ion intensities was performed with Progenesis QI™ Informatics for Proteomics software (Nonlinear Dynamics/Waters) and ProteinLynx Global Server (PLGS V3.0). HD‐MS^E^ parameters were set as follows: low energy threshold of 135 counts, elevated energy threshold of 30 counts, and intensity threshold of precursor/fragment ion cluster 750 counts. Chromatograms were automatically aligned by the Progenesis QI™ software, and those with alignment score ≥70% to the reference run were selected for further analysis. All spectra were manually checked following alignment, and additional vectors were added to improve the scoring. The percentage of rejected runs was <5%. To compare the controls to other subjects, we utilized the *between‐subject* design scheme of Progenesis QI™ software.

Database searches were carried out against UniProt‐SwissProt reviewed human database (release 2017_6, 48614 entries) with Ion Accounting algorithm as described (Mäkelä *et al*., 2016; Tikka *et al*., 2016). Following parameters were used: peptide and fragment tolerance: automatic, maximum protein mass: 500 kDa, min fragment ions matches per protein ≥7, min fragment ions matches per peptide ≥3, min peptide matches per protein ≥1, primary digest reagent: trypsin, missed cleavages allowed: 2, fixed modification: carbamidomethylation C, variable modifications: deamination of N/Q residues, oxidation of Methionine (M), and false discovery rate (FDR) <4%. Protein quantitation was performed entirely on nonconflicting *Homo sapiens* identifications, using precursor ion intensity data and standardized expression profiles. Protein was considered to be identified when it was present in all three replicates of the nano‐LC‐HD‐MS^E^ runs and within a study group when identification failed in less than in 33% of the participants (i.e., protein called present at least four of six participants within premenopausal group and at least seven of nine participants within postmenopausal group).

### Functional analysis of proteomics data

DEPs (*P* < 0.05 by ANOVA for all comparisons), which were quantified based on ≥2 unique peptides, served as inputs into IPA (Ingenuity Systems, Redwood City, CA; www.ingenuity.com). Prior to network and other functional analyses, the list of proteins was filtered to include only proteins with fold changes, FC greater than |1.5|. The IPA right‐tailed Fisher's exact test with B‐H multiple testing corrections was used to determine a *P*‐value of significance in all functional analyses.

### Functional validation of proteomics data

Primary human muscle cell line that was derived from the quadriceps muscle biopsy of a 5‐day‐old female infant (Edom *et al*., 1994) was kindly provided by Profs Moyly and Buttler‐Browne (INSERM, Paris, France). Proliferating mononuclear myoblasts were differentiated for 5 days to form multinuclear myotubes in E_2_ free environment, before exposing them to 10 nm E_2_, 100 nm E_2_ or mock for 6 h. All experiments were carried out in triplicate. Semi‐quantitative immunoblotting analysis was carried out to quantitate the protein expression of CUL4A, MT‐CO2, LDHA, ALDOA, and PKM. TUBA was used for normalization. The blots were scanned and quantified using Odyssey CLX Infrared Imager of Li‐COR and manufacturer's software. Cell culture mediums, fetal bovine serum, and antibiotics were obtained from Life Technologies, Inc. (Carlsbad, CA, USA) while insulin from Sigma‐Aldrich. CUL4A, MT‐CO2, and LDHA antibodies were purchased from Cell Signaling Technology (Danvers, MA, USA), ALDOA and PKM from Abcam (Cambridge, UK) and TUBA from Sigma‐Aldrich. As a secondary antibodies, anti‐rabbit IR Dye 800 or anti‐mouse IR Dye 680 (LI‐COR Biosciences, Lincoln, NE, USA) was used.

### Statistics

Statistical testing regarding phenotype variables and cell experiment was carried out with SPSS version 24 (IBM, Chicago, IL, USA). Shapiro–Wilk test and visual inspection of the normality plots were used to inspect normal distribution of the variables. Based on distribution, either independent samples *t*‐test or Mann–Whitney *U*‐test (in case of unrelated groups) or paired‐samples *t*‐test or Wilcoxon signed rank test (in case of twin pairs) were used.

## Accession numbers

The proteome data reported in this study are available in the Proteomics IDEntifications (PRIDE) data repository under accession number PXD006446 (Vizcaíno et al., 2016).

## Funding

This work was supported by Sohlberg's Foundation (E.K.L), Academy of Finland (J.K, V.K), and EFCP7 Collaborative Project MYOAGE (GA‐223576; S.S).

## Author contributions

E.K.L., J.K., U.M.K., S.S., and V.K. were responsible for human study design and experiments; M.L., R.S., and M.B. designed and conducted proteomic experiments; S.K. ran the Western blots; E.K.L. and M.L. conducted bioinformatics analyses; and E.K.L., R.S., and M.L. wrote the article. All authors contributed to the writing process and approved the final version of the article.

## Conflict of interest

The authors have declared no conflict of interests.

## Supporting information


**Table S1** List of all identified and quantified proteins in HD‐MSE experiments in the female muscle samples.

**Table S2** List of differentially expressed proteins (DEPs).

**Table S3** Protein lists used to create Fig. 1.

**Table S4** Affected canonical pathways and their contributing proteins in the female muscle.
